# Supplementary material for: A temporal classifier predicts histopathology state and parses acute-chronic phasing in inflammatory bowel disease patients
Source: Commun Biol. 2023 Jan 24;6:95. doi: 10.1038/s42003-023-04469-y (PMC9873918; doi:10.1038/s42003-023-04469-y)
Supplement: Supplementary file 2 — Supplementary Information [file 42003_2023_4469_MOESM2_ESM.pdf]

Supplementary Figures

Supplementary Figure 1: Overlap of differential splicing and differential expression trajectory genes

Supplementary Figure 2: Genes differentially expressed versus differentially spliced by fold change

Supplementary Figure 3: Gene biotypes by cluster

Supplementary Figure 4: VDJ saturation, burden and clonality at various acute colitis induction and recovery time-points

Supplementary Figure 5: Datasets and workflow of analysis

Supplementary Figure 6: Variance partition

Supplementary Figure 7: Depicts correlations between aggregate lymphocyte counts (binned into 'low', 'medium' and 'high') and normalized VDJ expression

Supplementary Data

Supplementary Data 1: VDJ clonal repertoire annotated by sample phenotypes (rows = number of clones detected in each sample, times number of samples clones were detected in)

Supplementary Data 2: VDJ summary statistics annotated by sample phenotypes (1 row per sample)

Supplementary Data 3: Time-point specific differential expression results from murine colitis models

Supplementary Data 4: Timepoint specific differential splicing results from murine colitis models

Supplementary Data 5: ILRL1 Differential splicing in human IBD

Supplementary Data 6: LAMA3 Differential splicing in human IBD

Supplementary Data 7: Disease_time_interaction_differential expression signature

Supplementary Data 8: Disease_time_interaction_differential splicing signature

Supplementary Data 9: MSCCR cohort demographics

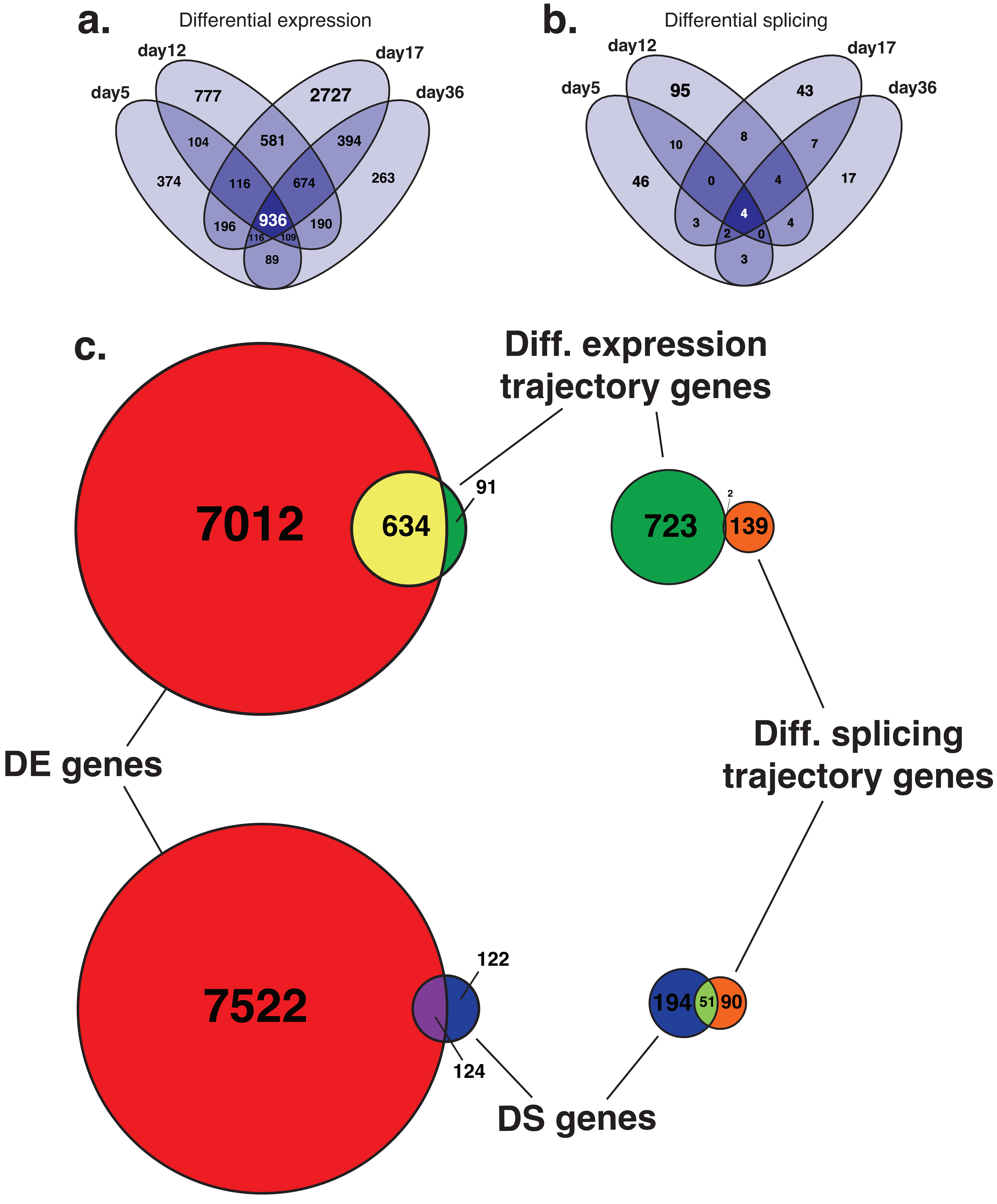
a.
Differential expression
day5
day12
day17
day36
777
2727
104
581
394
374
116
674
263
196
936
190
116
109
89
b.
Differential splicing
day5
day12
day17
day36
95
43
10
8
7
46
0
4
17
4
3
4
2
0
3
c.
Diff. expression trajectory genes
7012
634
91
723
2
139
DE genes
Diff. splicing trajectory genes
7522
122
124
194
51
90
DS genes

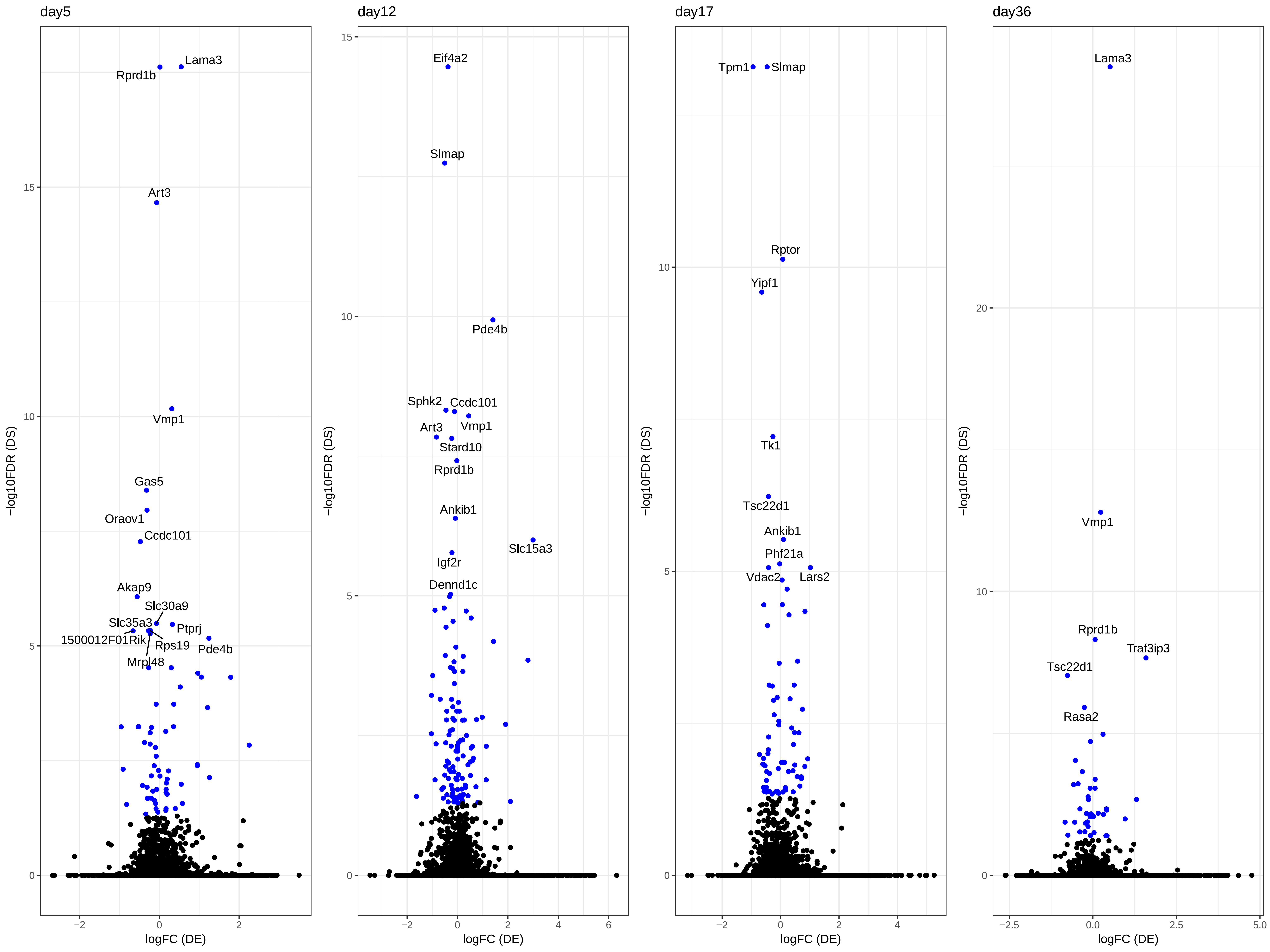
day5
day12
day17
day36
Lama3
Rprd1b
Art3
Vmp1
Gas5
Oraov1
Ccdc101
Akap9
Slc30a9
Slc35a3
Ptprj
1500012F01Rik
Rps19
Pde4b
Mrpl48
Eif4a2
Slmap
Pde4b
Sphk2
Ccdc101
Art3
Vmp1
Stard10
Rprd1b
Ankib1
Slc15a3
Igf2r
Dennd1c
Tpm1
Slmap
Rptor
Yipf1
Tk1
Tsc22d1
Ankib1
Phf21a
Vdac2
Lars2
Lama3
Vmp1
Rprd1b
Traf3ip3
Tsc22d1
Rasa2
−log10FDR (DS)
logFC (DE)

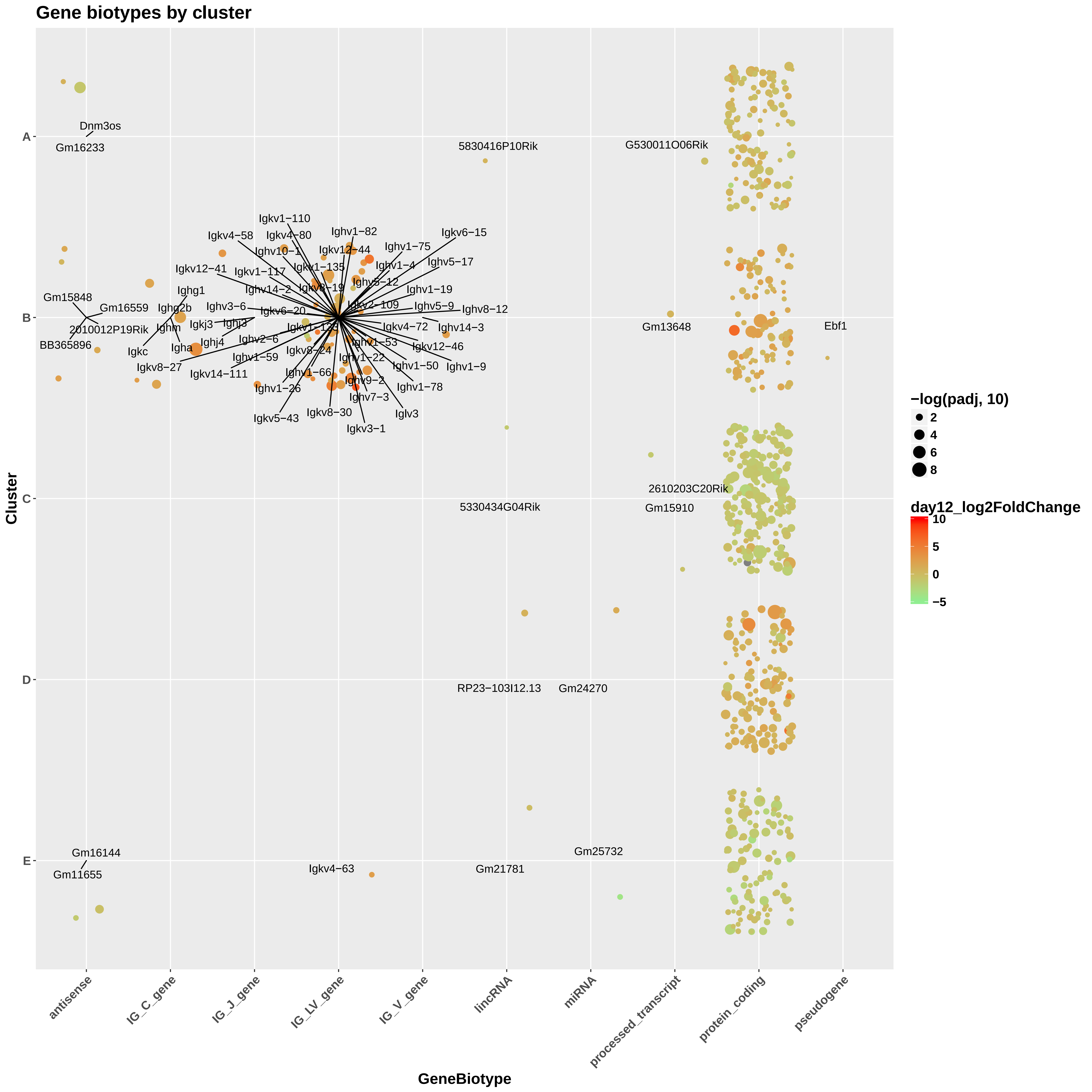

Gene biotypes by cluster
Cluster
A
B
C
D
E
GeneBiotype
antisense
IG_C_gene
IG_J_gene
IG_LV_gene
IG_V_gene
lincRNA
miRNA
processed_transcript
protein_coding
pseudogene
−log(padj, 10)
2
4
6
8
day12_log2FoldChange
10
5
0
−5
Dnm3os
Gm16233
5830416P10Rik
G530011O06Rik
Gm15848
Gm16559
2010012P19Rik
BB365896
Ighg1
Ighg2b
Ighm
Igha
Igkc
Igkv8−27
Igkj3
Ighj3
Ighj4
Igkv1−110
Igkv4−58
Igkv4−80
Ighv1−82
Igkv6−15
Ighv10−1
Igkv12−44
Ighv1−75
Igkv12−41
Igkv1−135
Ighv1−4
Ighv5−17
Igkv1−117
Ighv5−12
Ighv14−2
Igkv8−19
Ighv1−19
Ighv3−6
Igkv6−20
Igkv2−109
Ighv5−9
Ighv8−12
Igkv1−133
Igkv4−72
Ighv14−3
Ighv2−6
Ighv1−53
Igkv12−46
Ighv1−59
Igkv8−24
Ighv1−22
Ighv1−50
Ighv1−9
Igkv14−111
Ighv1−66
Ighv9−2
Ighv1−78
Ighv1−26
Ighv7−3
Igkv5−43
Igkv8−30
Iglv3
Igkv3−1
Gm13648
Ebf1
2610203C20Rik
5330434G04Rik
Gm15910
RP23−103I12.13
Gm24270
Gm16144
Gm11655
Igkv4−63
Gm21781
Gm25732

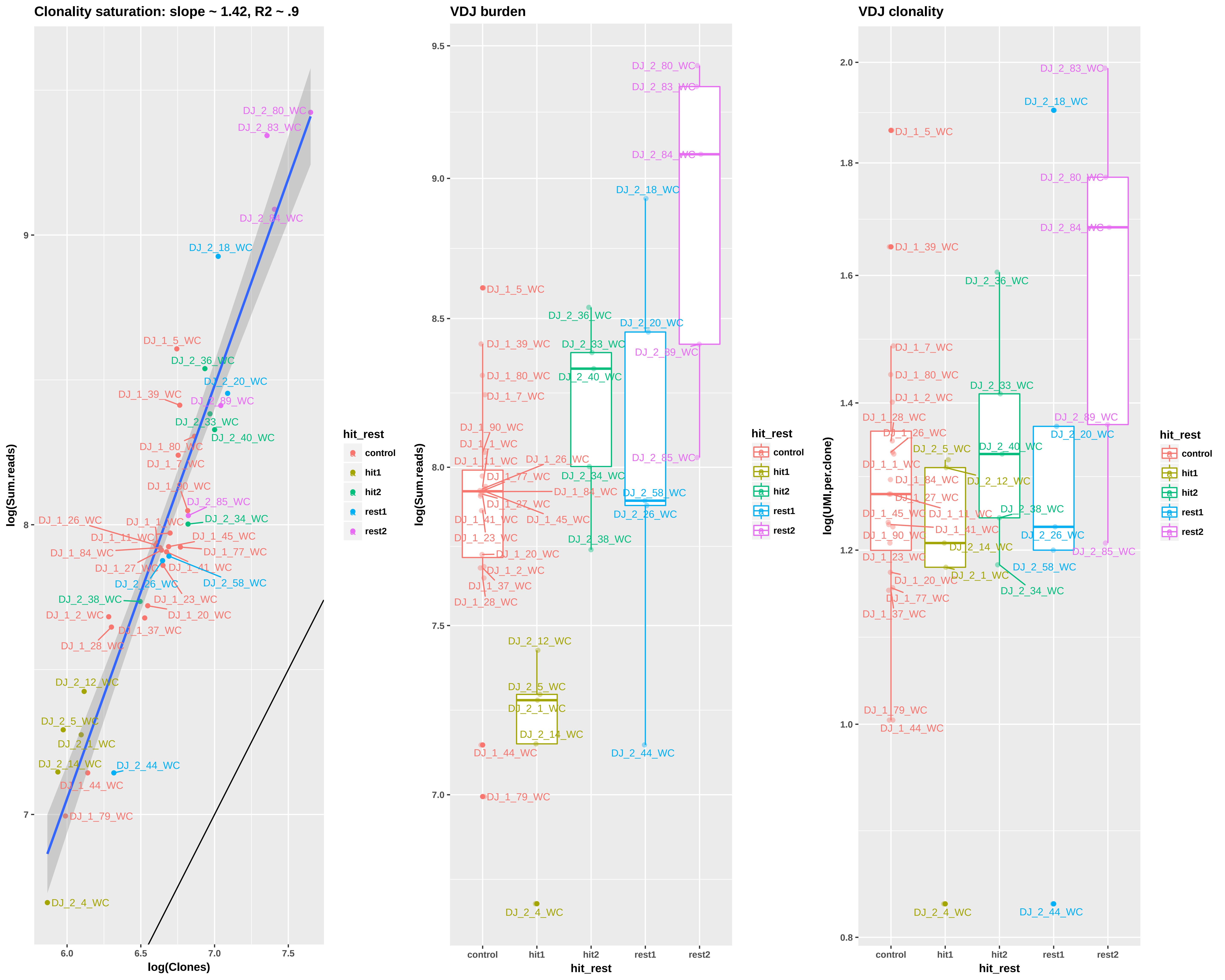
Clonality saturation: slope ~ 1.42, R2 ~ .9
log(Sum.reads)
log(Clones)
6.0
6.5
7.0
7.5
7
8
9
hit_rest
control
hit1
hit2
rest1
rest2
VDJ burden
log(Sum.reads)
hit_rest
7.0
7.5
8.0
8.5
9.0
9.5
control
hit1
hit2
rest1
rest2
hit_rest
control
hit1
hit2
rest1
rest2
VDJ clonality
log(UMI.per.clone)
hit_rest
0.8
1.0
1.2
1.4
1.6
1.8
2.0
control
hit1
hit2
rest1
rest2
hit_rest
control
hit1
hit2
rest1
rest2

Data Sets and Workflow

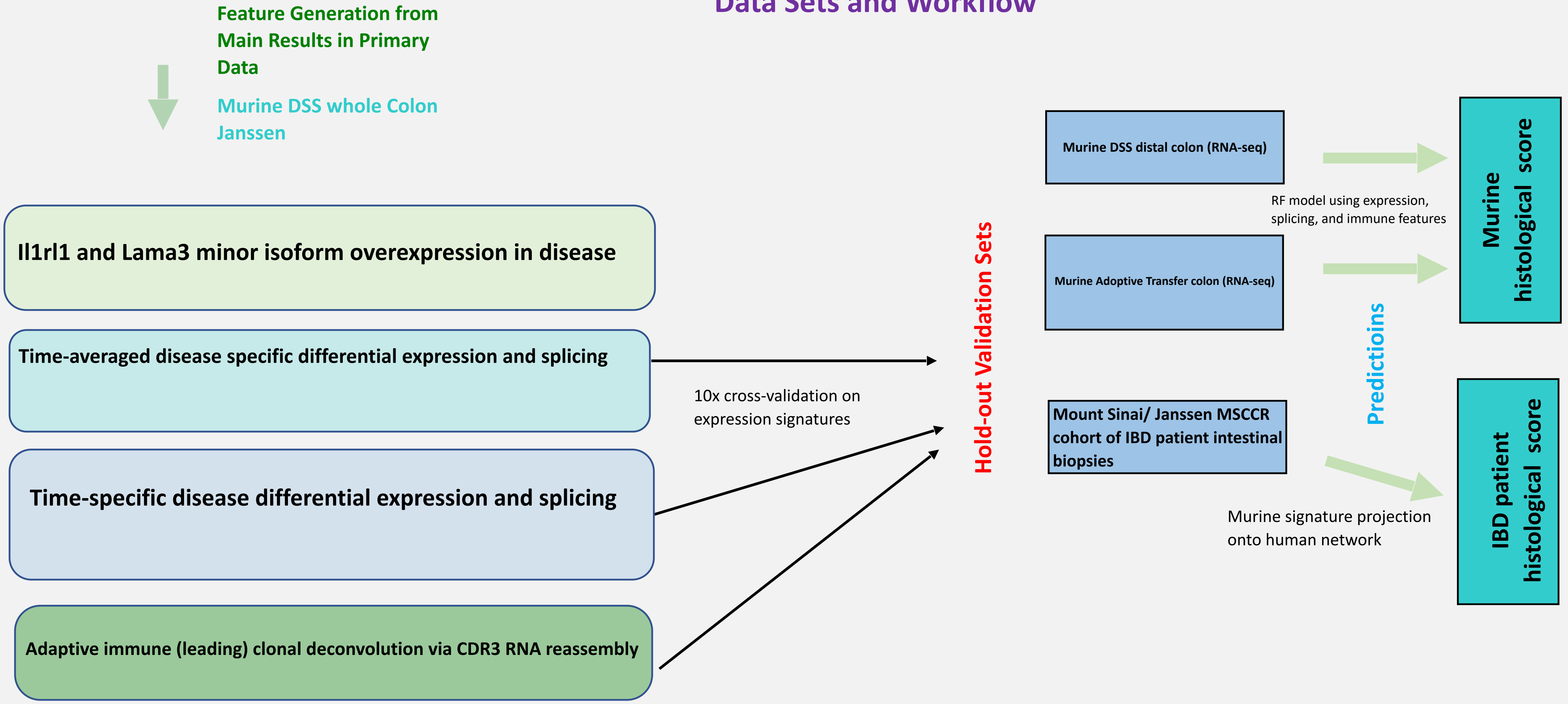

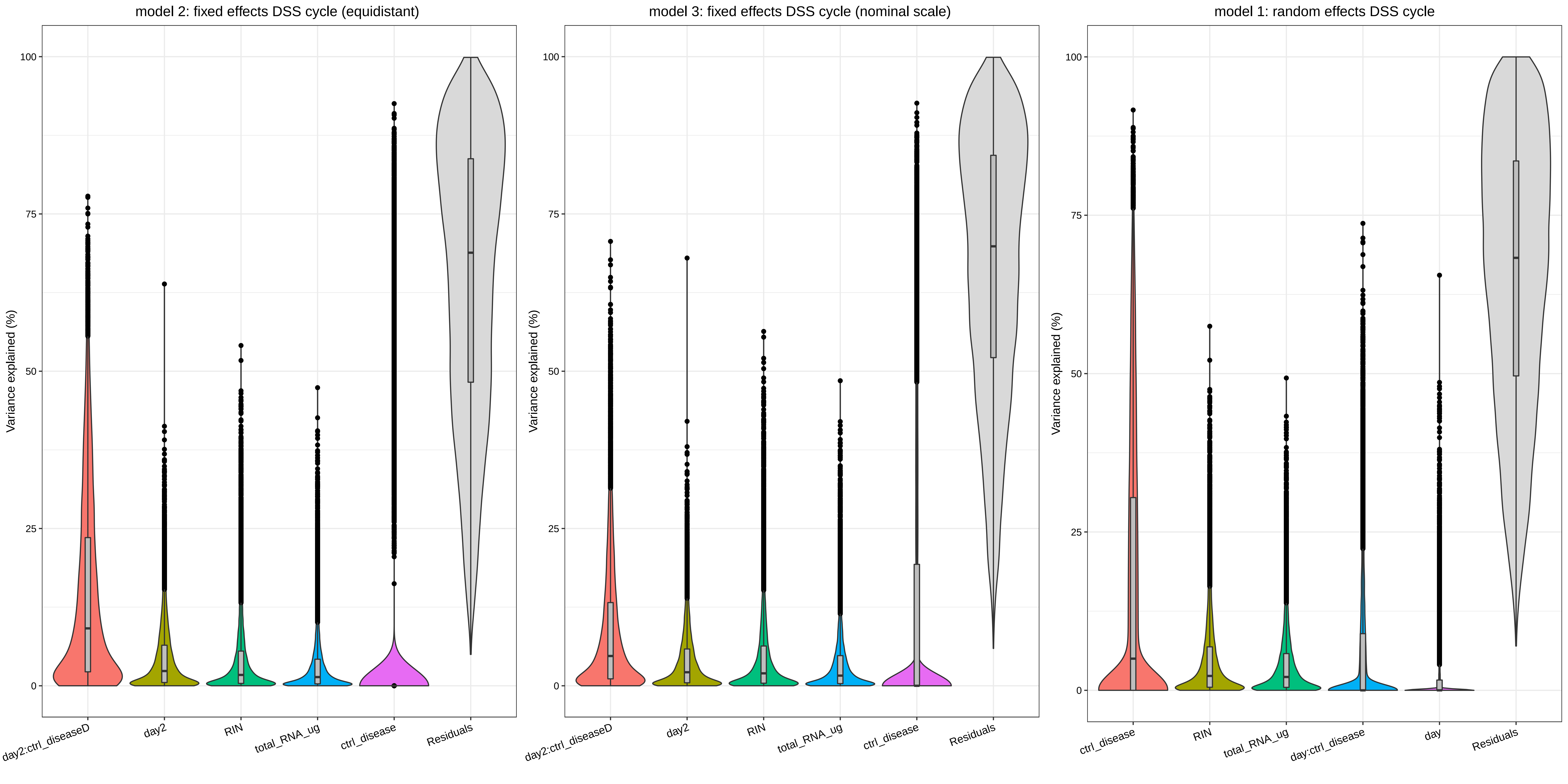

model 2: fixed effects DSS cycle (equidistant)
model 3: fixed effects DSS cycle (nominal scale)
model 1: random effects DSS cycle
Variance explained (%)
0
25
50
75
100
day2:ctrl_diseaseD
day2
RIN
total_RNA_ug
ctrl_disease
Residuals
day:ctrl_disease
day

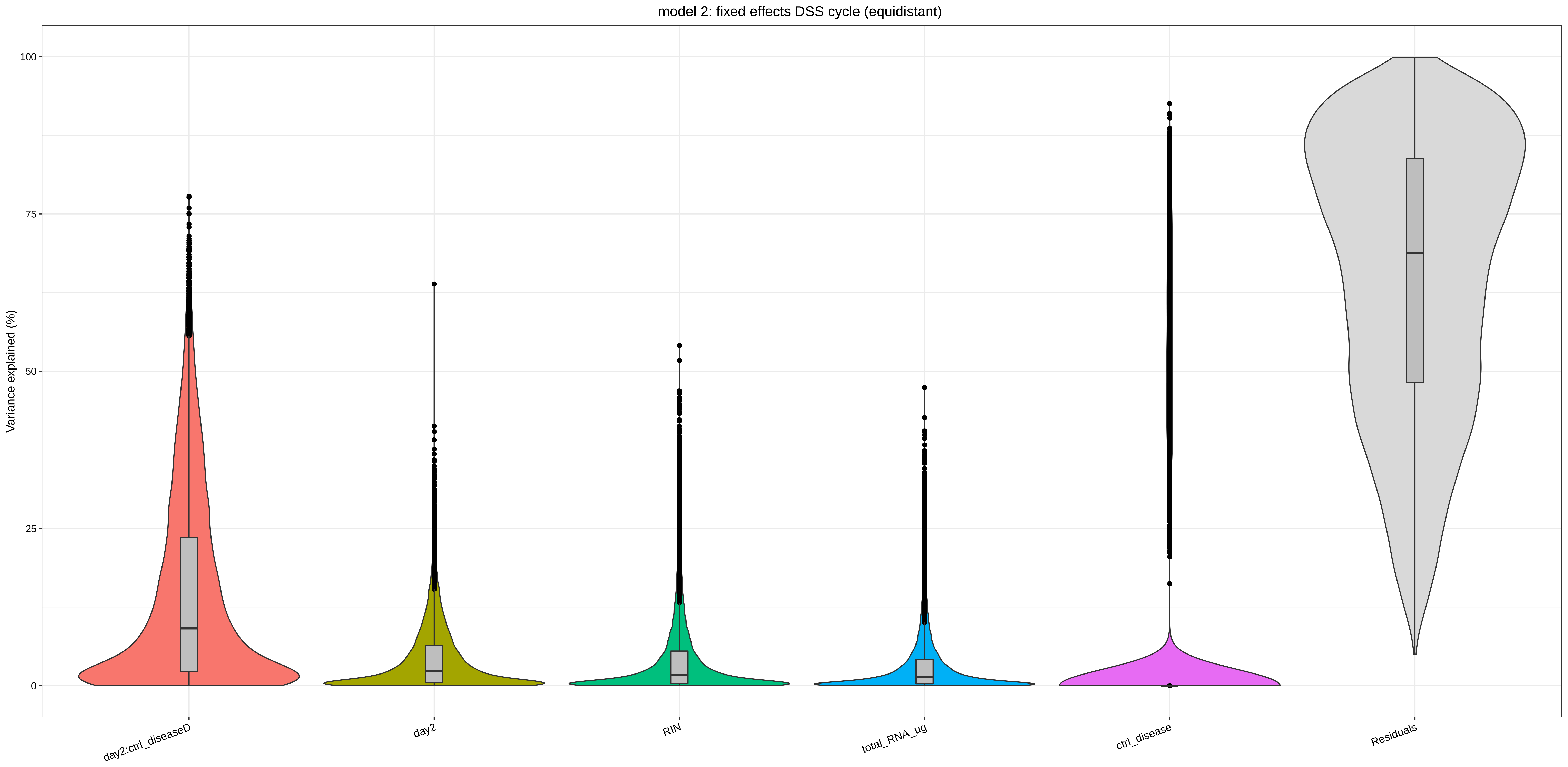
model 2: fixed effects DSS cycle (equidistant)
Variance explained (%)
100
75
50
25
0
day2:ctrl_diseaseD
day2
RIN
total_RNA_ug
ctrl_disease
Residuals

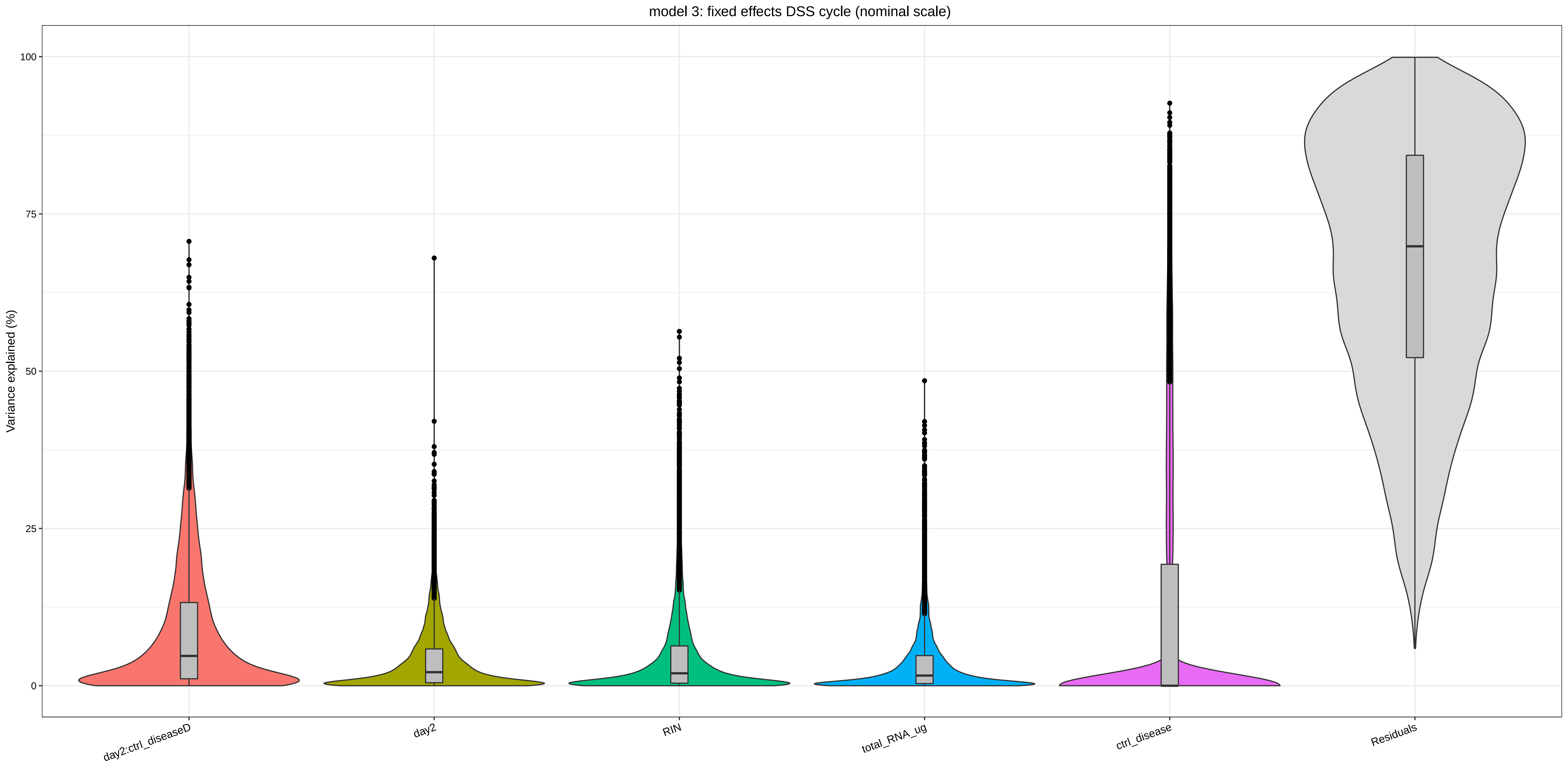
model 3: fixed effects DSS cycle (nominal scale)
Variance explained (%)
100
75
50
25
0
day2:ctrl_diseaseD
day2
RIN
total_RNA_ug
ctrl_disease
Residuals

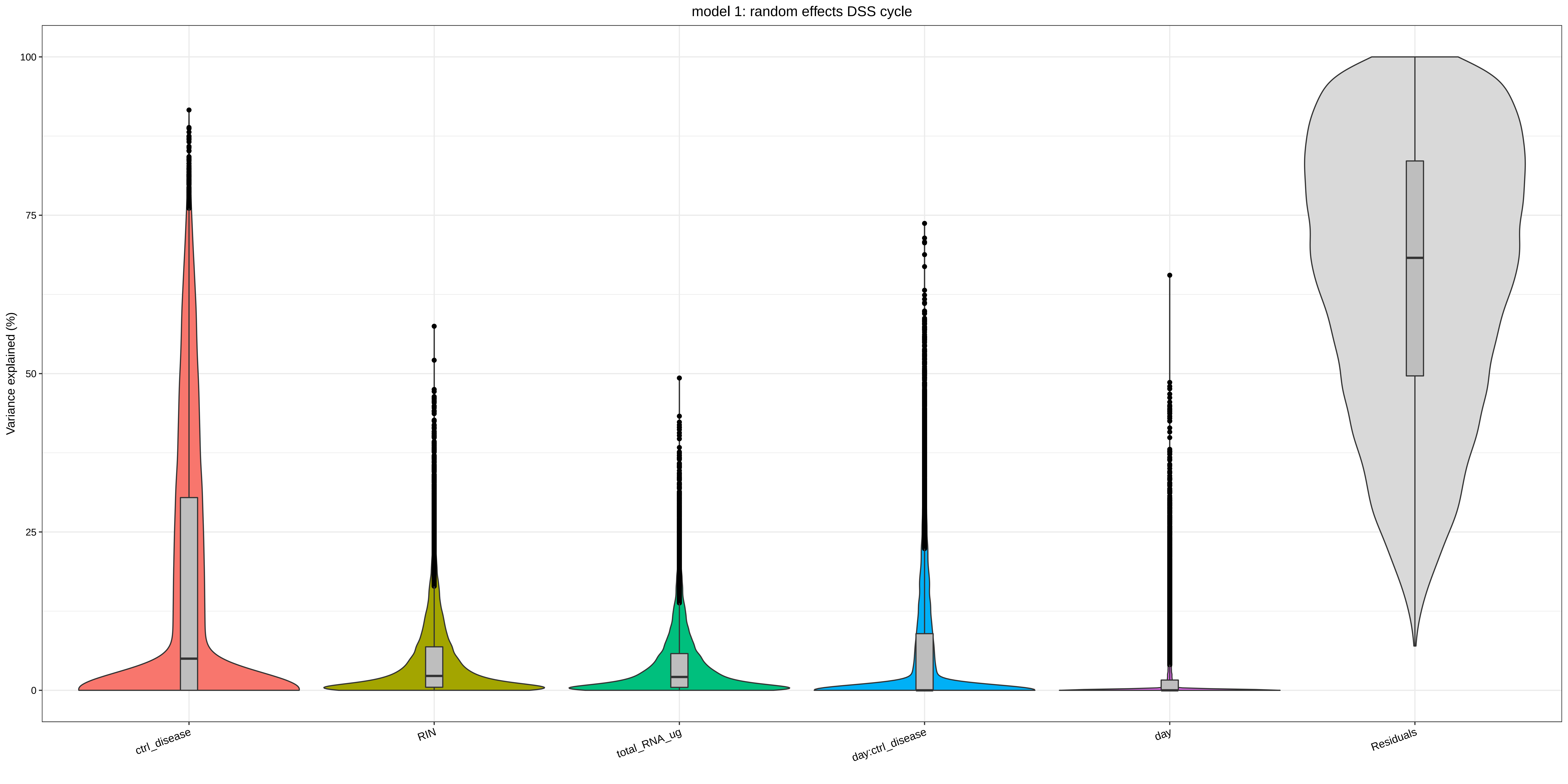
model 1: random effects DSS cycle
Variance explained (%)
100
75
50
25
0
ctrl_disease
RIN
total_RNA_ug
day:ctrl_disease
day
Residuals

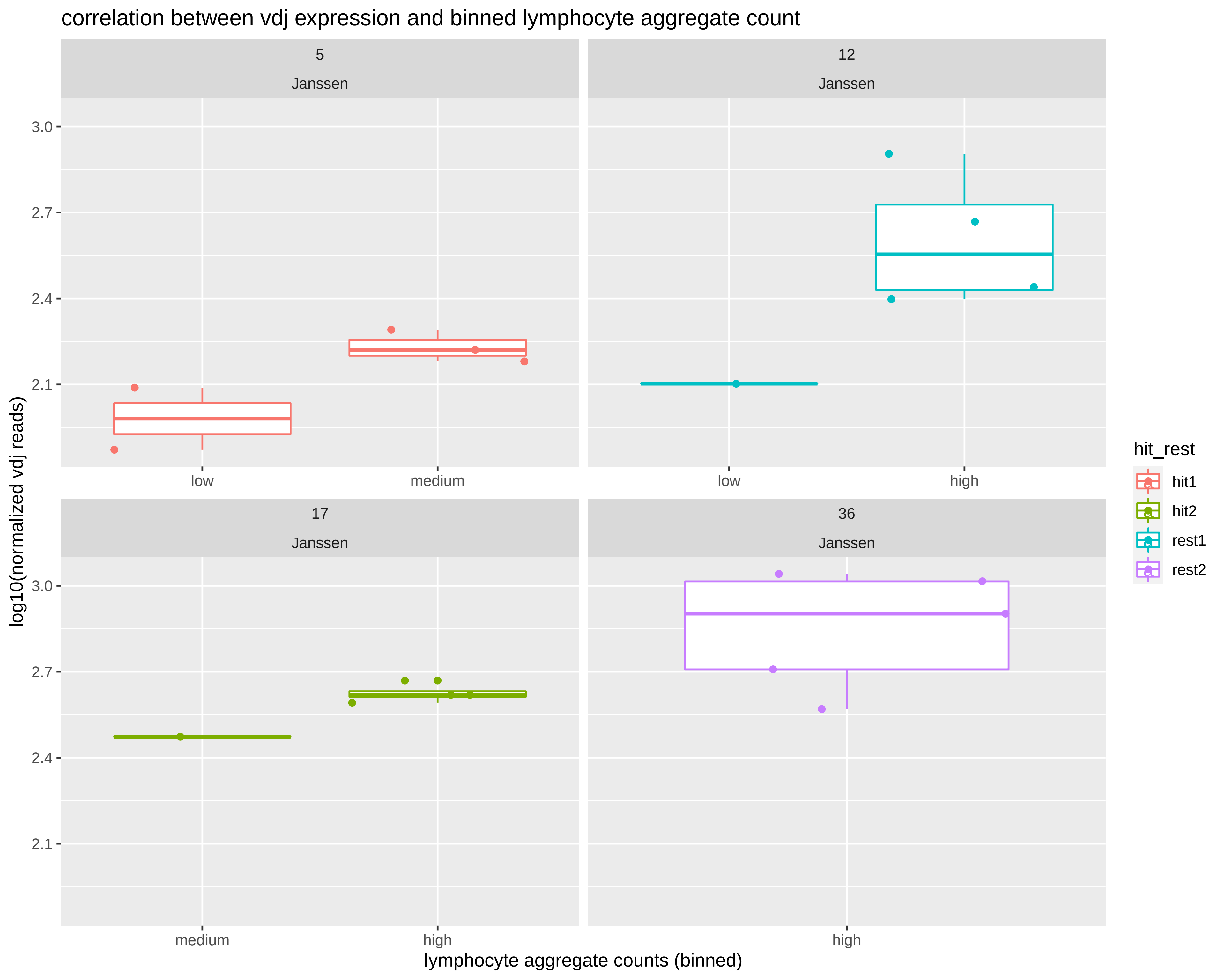
correlation between vdj expression and binned lymphocyte aggregate count
5
Janssen
12
Janssen
17
Janssen
36
Janssen
3.0
2.7
2.4
2.1
low
medium
low
high
medium
high
high
log10(normalized vdj reads)
lymphocyte aggregate counts (binned)
hit_rest
hit1
hit2
rest1
rest2
