# Supplementary material for: A temporal classifier predicts histopathology state and parses acute-chronic phasing in inflammatory bowel disease patients
Source: Commun Biol. 2023 Jan 24;6:95. doi: 10.1038/s42003-023-04469-y (PMC9873918; doi:10.1038/s42003-023-04469-y)
Supplement: Supplementary file 3 — Description of Additional Supplementary Files [file 42003_2023_4469_MOESM3_ESM.pdf]

Description of Additional Supplementary Files

File name: Supplementary Data 1
Description: VDJ clonal repertoire annotated by sample phenotypes (rows = number of clones detected in each sample, times number of samples clones were detected in).

File name: Supplementary Data 2
Description: VDJ summary statistics annotated by sample phenotypes (1 row per sample).

File name: Supplementary Data 3
Description: Time-point specific differential expression results from murine colitis models.

File name: Supplementary Data 4
Description: Timepoint specific differential splicing results from murine colitis models.

File name: Supplementary Data 5
Description: ILRL1 Differential splicing in human IBD.

File name: Supplementary Data 6
Description: LAMA3 Differential splicing in human IBD.

File name: Supplementary Data 7
Description: Disease_time_interaction_differential expression signature.

File name: Supplementary Data 8
Description: Disease_time_interaction_differential splicing signature.

File name: Supplementary Data 9
Description: MSCCR cohort demographics.

File name: Supplementary Code 1
Description: Code for analysis and pipelines in the study.
